# Demyelinating disorders in women: epidemiology, immunology, and clinical implications across MS, NMOSD, and MOGAD

**DOI:** 10.1007/s00415-026-13831-z

**Published:** 2026-04-27

**Authors:** Gabriel Ind, Zain Hashmi, Shivansh Ahuja, Tamara Fayad, Zaneh Kahook, Rumaiza Ahmad, Syeda Maryam Batool, Keziah Mariam Jiji, Hafsah Hudli, Shamera Hossain, Ali Lafi, Abdallah AbuJlambo

**Affiliations:** 1 GKT School of Medical Education, King’s College London, London, UK; 2 Department, King’s College Hospital NHS Foundation Trust, London, UK; 3 K S Hegde Medical Academy, NITTE University, Mangaluru, India; 4 Neurology Division, Saint George Hospital University Medical Center, Beirut, Lebanon; 5 Nova Southeastern University Kiran C. Patel College of Osteopathic Medicine, Fort Lauderdale, FL USA; 6 Baqai Medical University, Karachi, Pakistan; 7 Jinnah Sindh Medical University, Karachi, Pakistan; 8 Government Medical College, Kottayam, India; 9 Tbilisi State Medical University, Tbilisi, Georgia; 10 Bradford Teaching Hospital, NHS Trust, Bradford, UK; 11 Washington University of Health & Science, Ramallah, Palestine; 12 Department of ICU, Al-Shifa Hospital, Gaza, Palestine

**Keywords:** Multiple sclerosis, NMOSD, MOGAD, Sex differences, Females, Pregnancy

## Abstract

Multiple sclerosis (MS), neuromyelitis optica spectrum disorder (NMOSD), and myelin oligodendrocyte glycoprotein-associated disease (MOGAD) are the major types of demyelinating disorders of the central nervous system (CNS). Demyelinating disorders impact women disproportionately and frequently present during reproductive years. These conditions can cause significant neurological disability and psychosocial challenges, especially for women. Because they often present during reproductive years, clinicians frequently manage contraception, pregnancy, and the postpartum period alongside disease control. Sex-specific evidence, therefore, becomes especially important for treatment decisions. Despite this, sex-specific differences in epidemiology, immunopathology, clinical features, and therapeutic response remain inconsistently addressed in both clinical practice and research. In this review, we synthesize current evidence on the factors underlying the female predominance observed in MS, NMOSD, and MOGAD, and discuss the clinical consequences of these findings. We investigate the influence of sex hormones, X-chromosome–mediated immune regulation, and immunological changes associated with pregnancy and the postpartum period, as well as disease-specific mechanisms. We also examine how these factors affect diagnosis, prognosis, therapeutic decision-making, pregnancy management, and quality of life. Finally, we highlight important gaps in knowledge and underscore the necessity for a sex-informed approach to the diagnosis, management, and research of autoimmune demyelinating diseases.

**Supplementary Information:**

The online version contains supplementary material available at 10.1007/s00415-026-13831-z.

## Introduction

Demyelinating disorders are characterized by immune-mediated myelin sheath damage, resulting in impaired neuron conductivity and physiology [1]. Among the central nervous system (CNS) demyelinating disorders, the current literature highlights Multiple Sclerosis (MS), Neuromyelitis Optica Spectrum Disorders (NMOSD), and Myelin Oligodendrocyte Glycoprotein-Associated Disease (MOGAD) as the most common [1]. These conditions account for significant long-term disability, healthcare utilization, and socioeconomic costs [2–4]. MS is a neuroinflammatory disease of the CNS characterized by inflammatory demyelination with axonal transection. It affects millions of individuals globally, with an age-standardized prevalence of 22.2 per 100,000 [5]. It typically affects adults aged 20–40 years, which marks a critical period for educational, occupational, and reproductive activity [5, 6]. NMOSD is an immune-mediated inflammatory disorder of the CNS, most often linked to AQP4-IgG, in which astrocyte injury is primary and demyelination occurs secondarily [1], with an adult-onset prevalence ranging from 0.34 to 10 per 100,000 individuals across different geographic regions [7, 8].

Our literature search revealed a well-established female predominance in CNS demyelinating disorders, with an overall sex ratio of 3:1 in MS and 4.7:1 in NMOSD. While MS prevalence is more tied to geographical factors, NMOSD is directly linked to the presence of AQP4 autoantibodies [9]. Large registry studies across different countries further support this association [10, 11]. Females exhibit higher baseline immune activity on average, including increased antibody production, B-cell activation, and CD4 + T-cell responses [12, 13]. Estrogen within the female body modulates the immune system by enhancing B-cell survival and autoantibody production while also reducing B-cell apoptosis, particularly during higher estrogen states such as puberty and early pregnancy [12, 14].

Beyond immune function and sex hormones, a genetic explanation for this sex-specific incongruence has also been explored. Females possess two copies of the X chromosome, which harbor genes responsible for T- and B-cell activation [15]. Some of these genes can escape X chromosome inactivation, resulting in expression on both X chromosomes and leading to the increased immune system activity seen in females [16]. In males, testosterone facilitates the activation of Th2 cells, thereby protecting the myelin sheath from proinflammatory cytokines [17]. As mentioned above, sex hormones in women play a critical role in modulating the immune system and influencing disease activity. The importance of this role is illustrated by the fact that in MS, relapse rates typically reduce during pregnancy, especially in the third trimester [18–20], and then increase in the first three months postpartum; in NMOSD, pregnancy-related changes are less consistent, with several studies reporting increased relapse activity, particularly postpartum [21]. Women of childbearing age affected by these diseases face clinical implications relating to both their condition during pregnancy and, separately, the hazards associated with disease-modifying medications, including teratogenicity, fertility, and breastfeeding safety [22]. Furthermore, clinicians should recognize sex disparities regarding medication efficacy, adverse events, and disease progression [23, 24]. Although there is growing evidence regarding sex-related differences in CNS acute autoimmune disorders, existing findings remain fragmented and are inconsistent with clinical practice. Women predominantly exhibit a higher prevalence of MS and aquaporin-4-positive NMOSD, most frequently experienced during the onset of their reproductive years. These factors reveal a knowledge gap in diagnostic, prognostic, and therapeutic considerations that are inconsistently addressed in existing clinical guidelines. Moreover, limited sex-disaggregated reporting and prespecified sex-stratified analyses in randomized trials exacerbate the issue [25].

This narrative review will synthesize current information on the epidemiology, pathophysiology, and clinical considerations of CNS demyelinating disorders, specifically MS, NMOSD, and MOGAD. We intend to provide sex-specific data across various demyelinating illnesses and reproductive phases of life. This review seeks to enhance biology-driven clinical care and underscore critical areas where evidence is scarce.

### Methodology

We chose the narrative review approach to allow an integrated, topic-based discussion of immunopathological mechanisms, sex-based factors, and clinical implications that are not consistently captured within systematic reviews and meta-analyses. The literature search included PubMed, Cochrane Library, and Scopus, with searches focused on epidemiology, immunopathogenesis, sex and gender differences, pregnancy-related issues, and therapeutic implications across demyelinating disorders. We prioritized population-based studies, registry data, randomized controlled trials, systematic reviews, and recent high-quality observational studies. We excluded case reports, very small case series (< 5 cases), and conference abstracts without sufficient methodological detail. Where multiple sources addressed the same question, we gave greater weight to the most recent, methodologically robust, and clinically informative evidence. In addition, we conducted a dedicated literature search to specifically evaluate whether randomized controlled trials (RCTs) reported sex-specific or sex-stratified differences in treatment efficacy or safety in MS, NMOSD, and MOGAD. This targeted search was performed independently by two reviewers in PubMed and Scopus, using combinations of disease-related terms and keywords related to sex, gender, subgroup analysis, treatment response, efficacy, and safety. We restricted searches to clinical trials published between 2000 and 2026; both databases were searched on 17 January 2026. Titles and abstracts were screened independently by two reviewers against predefined eligibility criteria. Full texts of potentially relevant studies were then retrieved and assessed independently by two different reviewers for inclusion using the same eligibility criteria. Studies were included if they were randomized clinical trials evaluating therapeutic efficacy or safety in MS, NMOSD, or MOGAD and were ultimately assessed for whether they reported sex-disaggregated outcomes, sex-stratified analyses, or sex-by-treatment subgroup effects. Formal risk-of-bias assessment was not undertaken because this was a narrative review rather than a systematic review of treatment effects. Instead, study design, sample size, recency, methodological quality, and clinical applicability were considered when interpreting the literature. The search strategies for both databases are provided in Online Resource 1: Table 1.

## Epidemiology of demyelinating disorders in women

### Overall prevalence

In recent years, there has been an increase in the prevalence of demyelinating CNS disorders [8, 26]. According to the Atlas of MS, in 2025, over 2.9 million people worldwide were living with MS, a 30% increase from 2013 [26, 27]. Prevalence rates vary widely, from less than 10 per 100,000 population in low-income countries (e.g., African and Western Pacific countries) to over 130 per 100,000 in the United States and European countries [28].

In Argentina, Japan, and Sardinia, the relapsing–remitting type accounts for more than 80% of all MS cases. The proportion of primary progressive MS (PPMS) among individuals with MS ranges from 2 to 31%. Secondary progressive MS (SPMS) is most prevalent in Northern Europe, with the highest frequencies reported in Sweden. Both SPMS and PPMS are more commonly observed in men, indicating sex-based differences in MS disease course [29]. According to the Atlas of MS report, 85% of patients with MS have the relapsing–remitting type, 12% have progressive forms, and 3% have an unknown disease type [24]. Possible explanations for this increasing prevalence include greater reliance on MRI in the diagnostic criteria for MS, enhanced medical knowledge and clinical competency among medical staff, and improved survival rates due to improved management strategies [30].

In contrast, the overall NMOSD prevalence ranged from 0.34 to 10 per 100,000 population, indicating that this disorder is much rarer in comparison to MS [7, 8]. The 2006 Wingerchuk et al.’s diagnostic criteria for NMOSD included the presence of both optic neuritis and acute myelitis with 2/3 supportive findings. However, this was revised in 2015 by the International Panel for NMO Diagnosis (IPND) to broader, simpler criteria requiring at least one clinical criterion and positive serology for AQP4-IgG [4, 31]. Despite this change, prevalence remained unchanged, and incidence increased during this period, likely reflecting improvements in early detection and the expansion of disease categories to encompass the extensive NMOSD spectrum [4, 8, 31]. In their population-based nationwide studies, Hor et al. reported that the overall prevalence of MOGAD was 1.3–2.5 per 100,000 with an annual incidence of approximately 3.4–4.8 per million [32]. However, limitations in clinician awareness and a lack of standardized diagnostic criteria across different countries likely underestimate the true prevalence.

### Sex differences and age onset

**Fig. 1:**
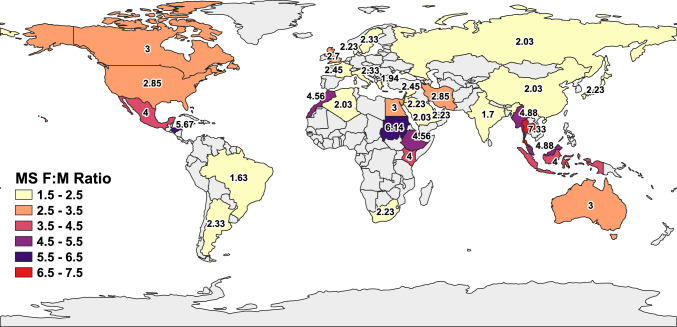
Global distribution of the female-to-male (F:M) sex ratio in multiple sclerosis

MS exhibits a strong global and regional female predominance; studies have found that the female-to-male ratio (F:M ratio) in MS is 2–3:1, with the majority of cases occurring in the relapsing–remitting form [33]. In NMOSD, female predominance is more pronounced, with a total F:M ratio of 4.7:1, rising to 9:1 in AQP4 antibody-positive patients [34]. Contrary to MS and NMOSD, the F:M ratio in MOGAD was much lower, with almost equal sex distribution ≈1:1 [9]. These differences suggest that sex-related biological factors, including hormonal and genetic influences, play a critical role across demyelinating disease subtypes in terms of incidence, prevalence, treatment, and prognosis. Females constitute roughly 66–78% of cases worldwide, including the Western Pacific, South-East Asia, the Americas, Europe, Africa, and the Eastern Mediterranean [26]. This female bias appears consistent worldwide, though its strength varies across populations and latitudes. Figure 1 illustrates the global geographic variation in the F:M ratio of MS prevalence, based on data from the Atlas of MS (2025). Colored regions indicate calculated ratios across 33 included countries, with gray regions indicating areas where data were not available for this narrative synthesis. According to the Atlas, the mean age of onset for MS in early adulthood is 30–32 years [35].

However, over the past 102 years, reports have noted an increase in late-onset MS (LOMS), defined as disease onset at age ≥ 50 years, from 2.6% in 1970 to 11.9% after 2010 [36, 37]. This trend may be attributed to modifications to the diagnostic McDonald criteria and to the widespread use of MRI since the 1990s, which has enabled more accurate diagnosis [36]. The mean age of onset in NMOSD is 41.7 years [34]. However, there is also variability in the median onset of NMOSD across different races, with Caucasians presenting with a median age of around 44 years, Asians presenting with a median age of nearly 36 years, and Afro-American/ Afro-European of 33 years [38]. A similar variability is observed in the NMOSD F:M ratio, ranging from ~ 3:1 in mixed U.S. cohorts to ~ 6:1 or higher in certain Asian cohorts [39].

**Fig. 2:**
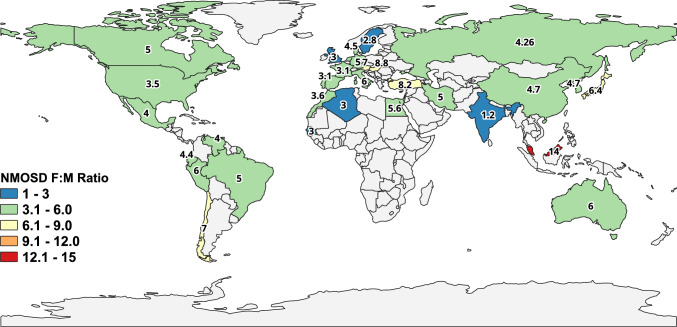
Global distribution of the female-to-male (F:M) ratio in neuromyelitis optica spectrum disorder (NMOSD)

A combination of genetic, sex-hormonal, and immunological factors could help explain variability in the F:M ratio [11, 34]. MOGAD demonstrates a broad age distribution with a median age of onset at around 28–30 years old across multiple nationwide cohorts [30]. Approximately 30% of cases occurred in pediatric populations, with clear seasonal variation in many regions [30]. In contrast to other demyelinating disorders, MOGAD demonstrates weak genetic associations, including limited links to HLA genes. It also shows no sex-ratio differences, with a nearly 1:1 FM ratio occasionally reaching 1.2:1 [32, 40, 41]. Figure 2 illustrates the global distribution of the F:M ratio in NMOSD across different countries, based on national numbers and a reliable systematic review and meta-analysis.

### Geographic and ethnic variation

The prevalence of acute demyelinating disorders varies by region, suggesting the influence of exogenous risk factors, including environmental causes such as Epstein-Barr virus (EBV) infection [42]. MS prevalence can be distributed geographically into three levels: high, medium, and low regions. MS demonstrates a well-established latitudinal gradient with higher prevalence observed in regions farther from the equator, including Northern Europe and North America. This distribution has been linked to reduced sunlight exposure and, consequently, lower vitamin D levels, which are believed to contribute to disease susceptibility. MS is more common among individuals of European ancestry, although its incidence is increasing globally, including in regions previously considered low risk. MS affects Caucasians most commonly, with lower vitamin D levels, geographic latitude, reduced sunlight exposure, childhood obesity, and smoking acting as associated increased risk factors [43]. BMI appears relevant as well, with overweight and obesity associated with higher MS risk [44]. Regions with high prevalence of MS are North American and European countries, with an estimated prevalence of > 100 [26, 45, 46]. The lowest prevalence was observed in Western Pacific countries and in Asian countries such as China, India, and Japan [26, 45].

When considering NMOSD, population-based studies report higher prevalence rates among African and Afro-Caribbean populations (approximately 10 per 100,000), followed by East Asian populations (around 3.4 per 100,000), while White populations in Europe and Scandinavia exhibit lower prevalence estimates of approximately 1.0–1.9 per 100,000 [7]. Similarly, African American populations in the United States show a higher standardized prevalence of approximately 8.44 per 100,000 compared to other ethnic groups [47]. These findings suggest that genetic susceptibility and population-specific immunological factors may play a key role in NMOSD distribution [48, 49]. Environmental risk factors such as ultraviolet exposure, Vitamin D status, infection exposure (e.g., EBV for MS), and latitude contribute to the substantial geographic variation seen in the epidemiology of demyelinating disorders [50, 51].

In MOGAD, prevalence shows modest variability across countries in Europe, Asia, the Americas, and the Caribbean, with no observed latitude gradient differences. Furthermore, ethnic variations have not been consistently reported across studies, particularly since the introduction of the international diagnostic criteria for MOGAD [32] (Online Resource 1: Table 2 includes references for NMOSD ratios).

### Incidence and trends

When discussing the burden of disease from demyelinating disorders, it is imperative to assess incidence and trends over time. Between 1990 and 2019, the global incidence of MS increased by 42%, from 41,854 to 59,345 [52]. Although this appears to be a significant increase in absolute terms, it was actually a small decrease when adjusted for age using the age-standardized incidence rate. The overall increase appears to be most pronounced in relapsing–remitting MS [53]. When broken down geographically, Northern Europe, North America, and Australia are commonly considered to have the highest incidence [52, 53], with other parts of Europe also reported [54]. However, this is not necessarily due to an actual increase in MS cases. With advancements in healthcare, we now have greater access to facilities with improved diagnosis and surveillance [52]. There has been an overall improvement in the quality of MS registries and databases, leading to a more accurate representation of MS cases rather than an actual increase [54]. The evolution of the diagnostic criteria for MS may also be responsible for the increased incidence observed, with the inclusion of patients previously diagnosed with ‘Clinically Isolated Syndrome’ and a shift toward MRI-based diagnosis [53, 54].

NMOSD has an estimated global incidence ranging from 0.03 to 0.73 per 100,000 people per year [7], and it is much rarer than MS, with fewer studies from which to draw reliable information. Interestingly, its regional incidence is more common in Afro-Caribbean and East Asian populations, which is in stark contrast to MS, highlighting that not all demyelinating disorders follow a standardized geographical distribution. Overall, the incidence of NMOSD has been quite stable over time, with no marked increase [7]. Where an increased incidence in NMOSD has been seen, it has been argued that this reflects improvements in antibody testing rather than new disease onset [7]. When considering MOGAD, it is important to note that it is a relatively new disease. Based on available data, the estimated prevalence of MOGAD is approximately 1.3–2.5 per 100,000 people, and the annual incidence is approximately 3.4–4.8 per million people [32]. Previously, it would have been diagnosed as NMOSD or MS rather than as a separate entity [7], which contributes to the lack of high-quality research from which to draw data. What sets MOGAD apart from the other demyelinating disorders mentioned in our review is its increased incidence in pediatric populations [39, 55]. Not much can currently be said about geographic or genetic predisposition due to the dearth of literature [32].

## Genetic factors

Genetic susceptibility plays a major role in the pathogenesis of autoimmune CNS demyelinating disorders. Genetic factors often interact with sex-specific immunological and hormonal factors, likely contributing to the observed female predominance in these conditions. In MS, the strongest and most consistent genetic association lies within the major histocompatibility complex (MHC), particularly the HLA-DRB1*15:01 allele, which confers an odds ratio of approximately 3 and accounts for a meaningful proportion of overall genetic risk [56]. Fine-mapping studies have also identified additional independent effects within the MHC, including class I alleles such as HLA-A and HLA-B, as well as non-HLA genes such as TNF and LST1, suggesting that multiple variants within this region may independently influence susceptibility [57]. Beyond the MHC, genome-wide association studies have identified more than 200 non-HLA susceptibility loci enriched in pathways related to T-cell activation, cytokine signaling, and B-cell function, further supporting the concept of MS as a polygenic immune-mediated disorder [57].

In NMOSD, the genetic contribution appears less clearly defined than in MS, although several HLA class II associations have been reported. These associations seem to vary across different populations and ancestries; for example, HLA-DRB1*03:01 has been associated with susceptibility in European, Brazilian, Afro-Caribbean, Mexican mestizo, and Indian cohorts [58]. In East Asian and Danish populations, other alleles, including HLA-DRB1*16:02, DRB1*08:02, DPB1*05:01, and DQB1*04:02, have all been identified as risk alleles, while HLA-DRB1*09:01 is thought to be protective [59]. In Chinese patients with AQP4-IgG-positive NMOSD, DQB1*05:02 and the DQB1*05:02-DRB1*15:01 haplotype have been linked to a markedly higher risk [60]. Likewise, in mixed Mexican and Colombian populations, certain HLA haplotypes and MHC SNPs have been reported more frequently, often reflecting Native American ancestral influences [58].

By contrast, MOGAD does not appear to have a strong or consistent HLA association. Instead, its pathogenesis may depend more on transient, trigger-dependent immune responses than on sustained inherited genetic risk. For example, large epidemiological studies have not identified a clear overall HLA association or consistent racial preponderance in MOGAD [32]. However, some studies have reported weak protective or subgroup-specific associations, including a possible protective effect of HLA-C*03:04 [61]. Notably, an important exception has been described in a Chinese pediatric-onset cohort: in this group, HLA-DQB1*05:02, DRB1*16:02, and the DQB1*05:02-DRB1*16:02 haplotype were associated with pediatric MOGAD, as well as with higher EDSS scores and increased relapse risk. In contrast, no clear HLA signal was identified in adult-onset disease [62]. Overall, the current evidence suggests that genetic predisposition is strongest and best established in MS, more heterogeneous in NMOSD, and least consistent in MOGAD.

## Pathogenesis

The typical causes of the discussed demyelinating disorders can be multifactorial. Genetic susceptibility accounts for 30–48% of overall disease risk, with the remaining susceptibility attributed to environmental factors [63, 64]. However, the pathogenesis of autoimmune CNS demyelinating disorders tends to follow a similar pattern: beginning with a loss of immune tolerance, progressing to peripheral immune activation, entering the CNS via blood–brain barrier (BBB) dysfunction, and ultimately causing tissue injury through disease-specific effector mechanisms.

### Loss of immune tolerance

Immune tolerance refers to the ability of the immune system to prevent destructive responses to self-antigens by autoreactive T and B lymphocytes through a process of negative selection. These immune cells are activated by molecular mimicry, such as that induced by EBV infection. Central immune tolerance is established during lymphocyte development through negative selection in the primary lymphoid organs: the thymus for T cells and the bone marrow for B cells. Peripheral immune tolerance is mainly mediated by regulatory T cells (Treg), which maintain immunological unresponsiveness to self-antigens [65]. When this control fails, high-affinity self-reactive T cells can escape from negative selection, become activated, and attack self-antigens such as myelin basic protein, proteolipid protein, and myelin oligodendrocyte glycoprotein, causing autoimmune CNS demyelinating disease [65, 66]. Genetic polymorphisms that alter immune regulation can influence these tolerance pathways and modify disease susceptibility. For instance, HLA class II molecules such as HLA-DR, HLA-DP, and HLA-DQ usually present antigens to CD4 + T helper cells and influence T-cell activation. In MS, HLA-DRB1*15:01 is associated with a threefold increased risk in individuals carrying at least one copy of this allele [67]. Inherited polymorphisms will alter antigen presentation and immune regulation, leading to loss of immune tolerance, escape of autoreactive T cells from deletion, and more readily activated autoreactive T cells due to more efficient presentation of myelin peptides [68]. In NMOSD, HLA-DPB1 induces activation of AQP4-specific CD4⁺ T helper cells and increases the risk of AQP4-IgG seropositivity. At the same time, MOGAD showed inconsistent HLA associations [49, 69].

Ultimately, genetic risk factors lower the threshold for pathogenic autoimmune activation by impairing two key immune tolerance checkpoints: thymic deletion of autoreactive immune cells and the weakening of peripheral regulatory circuits.

### Peripheral immune activation

Following the loss of immune tolerance, the next component of the pathological sequence begins with the activation of the peripheral immune system via environmental triggers acting on a genetically susceptible immune system. As mentioned above, common exogenous and lifestyle factors include EBV infection [70], vitamin D deficiency [71], and smoking [72]. Among genetically susceptible individuals, these conditions promote activation of autoreactive CD4 + T cells (particularly Th1 and Th17 subsets) and B cells [63]. It has been reported that decreasing the level of 25-OH Vitamin D doubles the risk of MS [73], and lower baseline 25-OH Vitamin D levels are also associated with a higher risk of disability progression and brain volume atrophy [74]. In addition, living at higher latitudes is associated with decreased exposure to ultraviolet radiation (UVR) and, consequently, reduced Vitamin D synthesis, which is linked to a higher risk of MS [75]. As a result of impaired immune tolerance, autoreactive CD4 + T cells will be activated in the peripheral immune system, which is consistent with the outside-in model of pathogenesis of MS. These activated T cells differentiate into Th1 and Th17 predominantly, which are considered to be the key factors of disease initiation [76]. Th1 cells secrete interferon gamma, which upregulates HLA class I and class II molecules, increasing the antigen-presenting capacity of macrophages, astrocytes, and microglia. Similarly, Th17 cells produce IL-17, a major contributor to inflammation that promotes endothelial dysfunction by generating reactive oxygen species, thereby facilitating early disruption of the blood–brain barrier (BBB) and the entry of immune cells into the central nervous system [76].

### Blood–brain barrier disruption (outside-in hypothesis)

The next step in the pathogenesis of demyelinating disorders relates to linking peripheral immune dysregulation to the development of inflammation in the central nervous system. The blood–brain barrier (BBB) prevents proinflammatory cytokines from entering the CNS, which can induce inflammation. In demyelinating disorders, disruption of the BBB permits the migration of autoactivated CD4 + T helper cells (Th1 and Th17), along with proinflammatory cytokines, into the CNS, where they initiate and propagate inflammatory demyelination. This process leads to upregulation of endothelial adhesion molecules, such as vascular cell adhesion molecule-1 (VCAM-1) and intercellular adhesion molecule-1 (ICAM-1), which facilitate leukocyte adhesion and transmigration across the BBB. Immune cells then initiate localized inflammation targeting the myelin sheath, oligodendrocytes, and astrocytes [77]. In NMOSD and MOGAD, the leaky BBB allows AQP4-IgG and MOG-IgG specific antibodies to enter the CNS and initiate disease activity [76]. BBB dysfunction in demyelinating disease is considered to be a biological switch that turns autoimmunity into neurological and clinical disease. This becomes notable during the clinical relapses and corresponds radiologically to gadolinium-enhancing lesions on MRI, underscoring the clinical relevance of imaging [78]. Treatment strategies targeting adhesion molecules, such as VLA-4, including the monoclonal antibody Natalizumab, are used to block immune cell movement across the BBB into the CNS [79].

### Effector mechanisms of autoimmune demyelinating CNS disorders

Although all the disorders we have discussed involve demyelination, the mechanisms underlying demyelination vary. They are all caused by an interplay between adaptive immunity and effector systems, involving T cells, B cells, macrophages, granulocytes, and the complement system [80]. In MS, myelin damage and neuronal injury are mainly driven by cell-mediated T cell immunopathology, particularly CD8 + cytotoxic cells and Th1 and Th17 CD4 + lymphocytes [80]. In addition to T-cell–mediated mechanisms, B cells play a central role in MS pathogenesis. B cells contribute through antigen presentation to T cells, secretion of pro-inflammatory cytokines such as IL-6 and TNF-α, and differentiation into plasma cells that produce intrathecal immunoglobulins. The presence of oligoclonal bands in cerebrospinal fluid reflects chronic B-cell activation within the CNS. Furthermore, antibodies may contribute to demyelination through complement activation and opsonization, amplifying tissue injury. The clinical efficacy of B-cell depleting therapies, such as anti-CD20 monoclonal antibodies, further supports the central role of B cells in MS [63]. Although MS is often viewed as mainly cell-mediated, antibodies and complement can contribute in some lesions, and long-term disability relates more to cumulative neuroaxonal injury than to any single effector pathway [81, 82].

In contrast, NMOSD is a primary astrocyte-targeting autoimmune disease against aquaporin-4 (AQP4) water channels in astrocyte end-feet at the BBB. The peripherally generated AQP4-IgG1 binds to astrocytes rather than oligodendrocytes, and subsequent robust activation of the classical complement pathway causes astrocytopathy, culminating in indirect injury to oligodendrocytes. The activation of macrophages ultimately leads to severe demyelination, necrosis, and axon injury. This effector mechanism helps explain the severity of attacks and the incomplete recovery seen in NMOSD [80, 83].

In contrast to both, MOGAD has a unique effector mechanism driven by an IgG-1 autoantibody targeting MOG, which is located in the outermost layer of myelin. This mechanism induces inflammatory demyelination without astrocytic injury in comparison to NMOSD [84]. MOG-reactive CD4 + T cells are activated in the meningeal spaces and produce Th17 cytokines that damage the BBB, allowing access of MOG-IgG1 antibodies and the opsonization of myelin, while preserving astrocytes [84]. Damage in MOGAD occurs via both the antibody and complement systems; however, complement activation is less pronounced than in NMOSD [84, 85]. The pathological features include perivenous demyelination and relative axonal preservation, with a higher remyelination potential, consistent with MOGAD’s often monophasic or relapsing, but steroid-responsive, nature [86]. The variation in the mechanism of injury has profound implications for the management of these disorders. By better understanding the processes underlying MS, NMOSD, and MOGAD, we can identify critical points and develop targeted therapies. For example, in NMOSD, understanding the mechanisms underlying demyelination has led to breakthroughs in complement inhibition and in B‑cell–depleting or IL-6-blocking therapies [87]. Concurrently, this understanding also gives us a unique insight into the epidemiology of demyelinating disorders.

### Disease-specific target antigens and immunopathology

Based on the above, the target cells for each disorder highlight an important difference in their pathophysiology. In MS, myelin basic protein (MBP), myelin-associated glycoprotein (MAG), and proteolipid protein constitute a multilamellar myelin sheath around the axons and neuron cell bodies and act as the target of circulating CD4 + T cells, resulting in chronic inflammatory demyelination with variable axonal loss and neurodegeneration [80, 87]. In contrast, NMOSD is a primary astrocytopathy mediated by pathogenic IgG1 antibodies directed against aquaporin-4 (AQP4) water channels located on astrocytic end-feet at the blood–brain barrier, leading to complement-dependent cytotoxicity; AQP4 receptors predominate in the astrocyte foot processes at the BBB. NMOSD can also be diagnosed in patients who are seronegative for AQP4-IgG; notably, in one well-characterized cohort, 42% of AQP4-IgG-negative NMOSD patients were MOG-IgG positive [83]. MOGAD is characterized by antibodies targeting the transmembrane protein MOG, a marker of mature oligodendrocytes. This process occurs in the outermost layer of the CNS myelin sheath and is involved in cell adhesion, microtubule stability, and receptor function. Although this forms a target cell in MS as well, it is more characteristic of MOGAD [84]. Although antibodies against MOG are central to MOGAD, they are generally absent in classic MS. Rarely, MOG-IgG may be detected in patients initially diagnosed with MS, highlighting the importance of accurate antibody testing for proper diagnosis and treatment selection. Unlike MS, MOGAD is often responsive to corticosteroids, IVIG, or plasma exchange, and treatment strategies differ from B-cell-targeted therapies commonly used in MS [88].

### Sex-specific immunopathological considerations

Demyelinating disorders have a particular relevance to women [37, 77], and this epidemiology can be explained by analyzing how sex differences can facilitate different aspects of these disorders. Sex hormones appear to influence disease prevalence and course, and these disorders are most common during the reproductive years [89]. Females have stronger innate and adaptive responses, with higher CD4 T-cell, B-cell, and antibody activity, but relatively fewer natural killer cells and monocytes [90, 91]. However, it is interesting to note that although women more strongly exert their immunity through myeloid pathways, men tend to do so through lymphoid or adaptive pathways. This immunological difference may explain why there is a higher incidence of demyelinating disorders in women; however, progression risk is much higher in men [92]. Female oligodendrocytes tend to upregulate antigen‑presentation and mitochondrial repair programs, explaining the differences in disease severity observed [93]. In MS, estrogen and progesterone are found to be protective in nature as they have been shown to promote remyelination and reduce inflammatory reactions [93, 94]. These effects are illustrated by pregnancy and hormonal contraception, where increased levels of sex hormones enhance oligodendrocyte survival and regeneration [89, 93]. With these relationships, it appears as though demyelinating disorders are not only more common in women but are also impacted by hormonal changes specific to the sex, and a better understanding of this relationship could help mitigate the morbidity and disability associated with them. Additionally, research shows that estrogen enhances humoral and Th1 and Th17 responses, whereas androgens and progesterone exert immunosuppressive effects. The impact of this can be seen in MS, where we often see pregnancy-related remission and a postpartum exacerbation [89]. In NMOSD, there is a weak connection between progesterone and disease severity. It has been hypothesized that progesterone in women can aggravate NMOSD presentation, and potentially counter-intuitively lead to decreased bioavailability of progesterone and other sex hormones [95].

### Pathophysiology-driven therapeutic implications

**Fig. 3:**
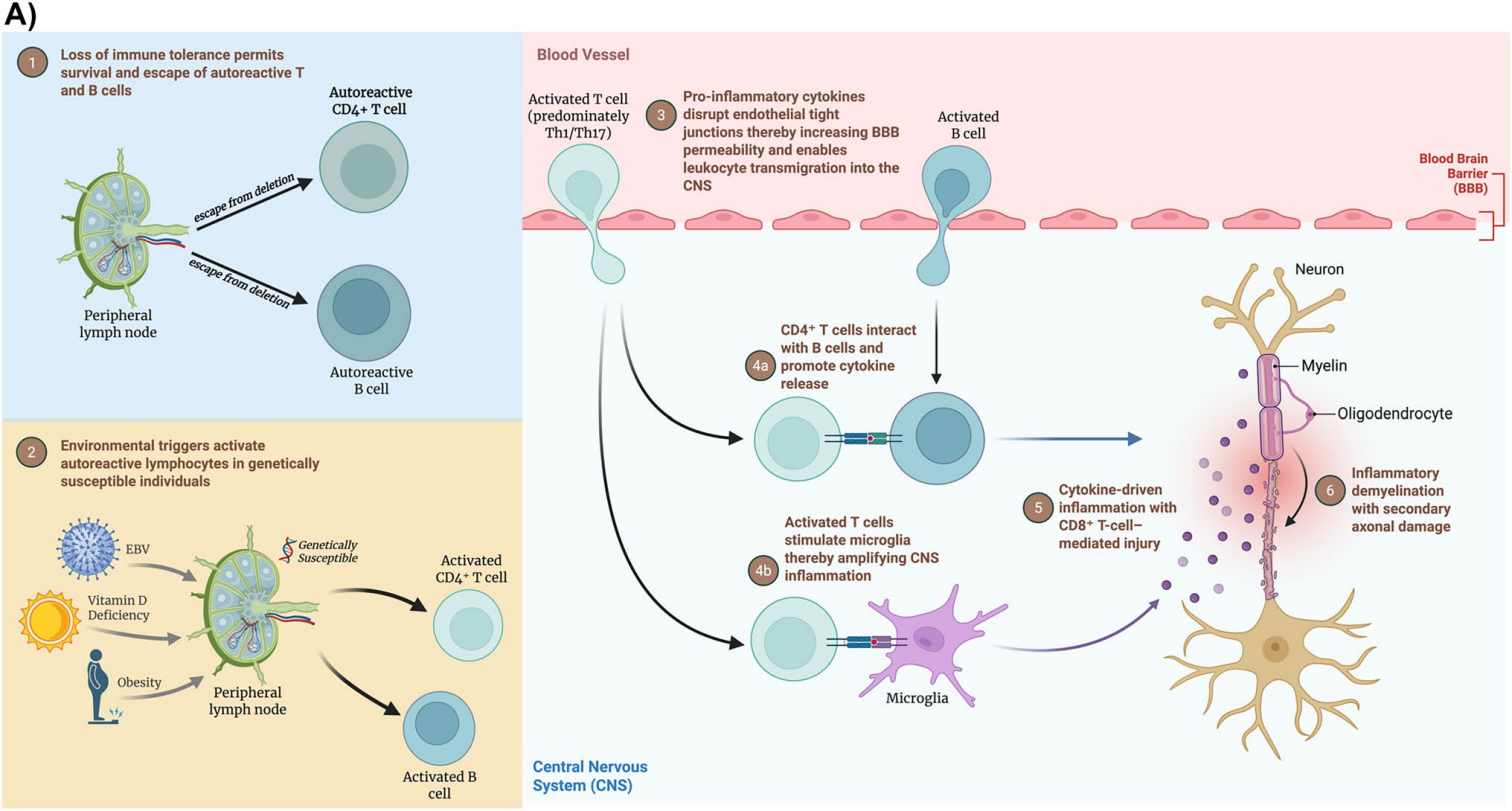

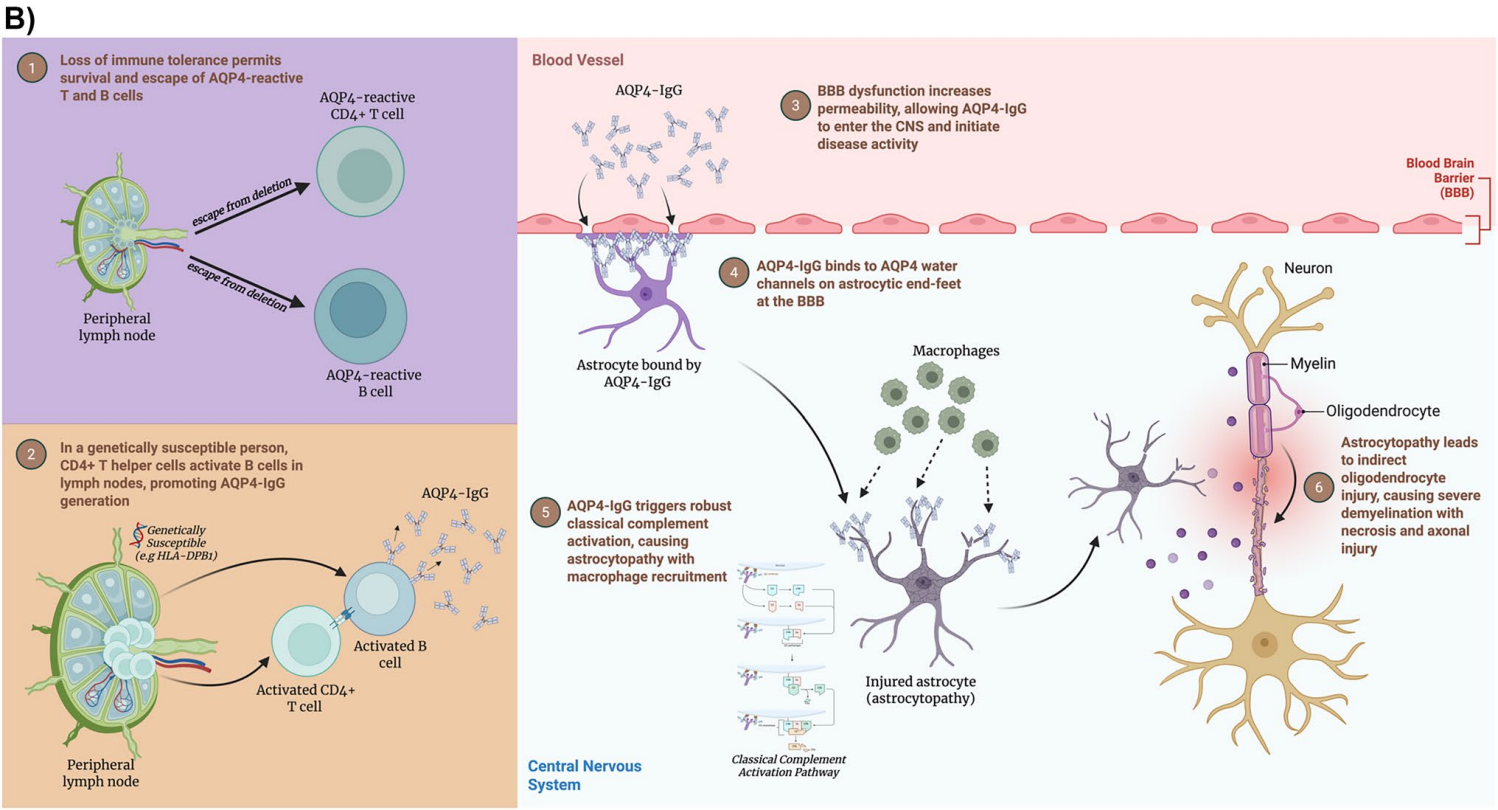

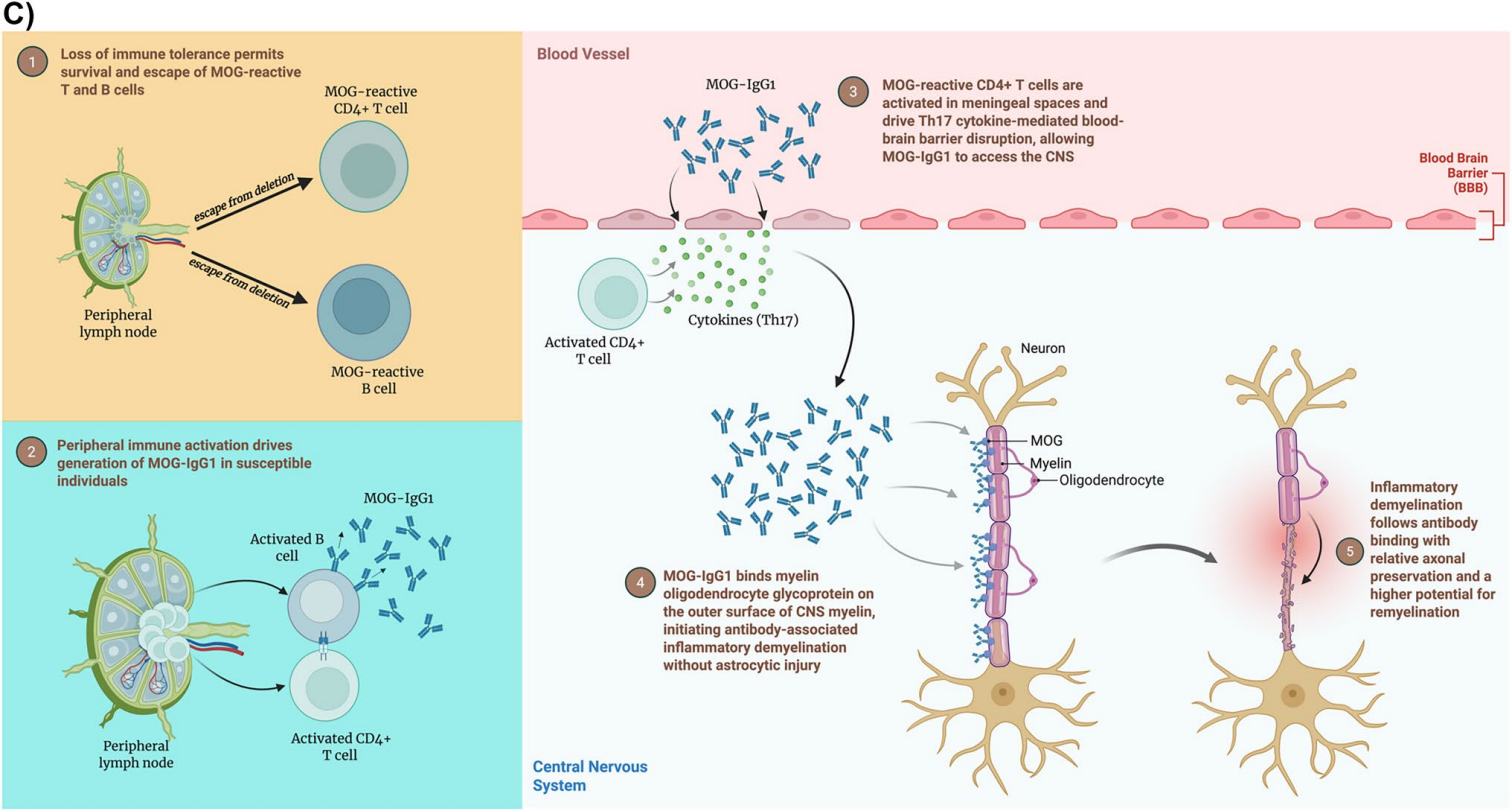
**A** Illustration of pathogenesis of multiple sclerosis (MS). **B** Illustration of pathogenesis of neuromyelitis optica spectrum disorder (NMOSD). **C** Illustration of pathogenesis of myelin oligodendrocyte glycoprotein antibody-associated disease (MOGAD). (**A**–**C** created with BioRender.com)

Understanding disease-specific mechanisms has directly informed therapeutic strategies. MS treatments target multiple pathways of immune dysregulation. While historically most disease-modifying therapies (DMTs) focused on T-cell activation and trafficking, B-cell-depleting therapies such as ocrelizumab and rituximab have demonstrated significant efficacy in reducing relapse rates and disease activity, highlighting the critical role of B cells in MS pathogenesis. In contrast, NMOSD therapies focus on B-cell depletion, IL-6 inhibition, and complement blockade, reflecting their antibody-mediated nature [18]. Figure 3A–C schematically illustrates the key stages of pathogenesis in MS, NMOSD, and MOGAD, respectively.

## Clinical implications

### Diagnostic implications

Sex-specific immunological factors influence both disease prevalence and diagnostic accuracy in demyelinating CNS disorders. The female predominance in these disorders is partly explained by a more active adaptive immune response in women. Women have higher CD4 + T cell counts and greater B-cell activity, supporting increased antibody production. These differences are influenced by sex hormones (e.g., estrogen) and by immune-regulating genes, which sex chromosomes may also modulate. FOXP3, TLR7/8, CD40L, and BTK all lie on the X chromosome, resulting in higher immune gene dosage in homozygous individuals (XX in women) and predisposing to stronger adaptive immunity [90, 91]. This suggests that factors beyond reproductive hormones contribute to disease susceptibility, including genetic and epigenetic mechanisms linked to the X chromosome, such as FOXP3 methylation and microRNA expression [89]. As established, MS is the most prevalent of the autoimmune demyelinating disorders. This prevalence can contribute to premature closure of a diagnostic process for MS when women present with optic neuritis or transverse myelitis, failing to consider NMOSD and MOGAD, particularly when specific antibody testing is not requested, or returned in a timely manner [96]. This creates a diagnostic gap where women are overdiagnosed with MS and men are underdiagnosed [9]. On the other hand, relying on clinical reasoning and neuroimaging (MRI) to diagnose the NMOSD and MOGAD makes those disorders easily overlooked as MS or diagnosed late due to a lack of specific antibody testing [96].

### Disease course and prognosis

There is a double peak of the age of onset in women who have MS. Interestingly, younger women are more likely to experience a relapsing–remitting (RRMS) and secondary progression disease (SPMS) type when compared with men, who are more likely to develop the primary progressive phenotype of MS (PPMS). Regarding the clinical presentation, women more commonly experience optic neuritis and sensory symptoms; however, men mainly present with motor symptoms [97]. On the other hand, late-onset MS in women is more frequently associated with a primary progressive phenotype and is linked to worse disability outcomes [89, 93, 98]. Women experience higher rates of relapse, as seen in the case of highly aggressive MS, where the F:M ratio is about 3.3:1. Men tend to accumulate disability more rapidly with poor relapse recovery, and progress earlier to secondary progressive MS with greater neurodegeneration. In addition, a greater cognitive impairment is seen in women [89, 93, 94]. However, for PPMS, there were no differences between women and men in disability progression [97].

### Therapeutic implications

Treatment of autoimmune demyelinating disorders should target specific effector mechanisms for each disease separately rather than relying on clinical features alone. In MS, disease-modifying therapies target multiple pathways of immune dysregulation. Sex-specific factors shape relapse rates, clinical course, and treatment responses; however, clear sex-stratified therapeutic algorithms are still lacking. A recent systematic review found no consistent evidence of sex-specific differences in response to MS-DMT due to a small sample size and a lack of subgroup analyses by sex [99]. In terms of safety, women are more likely to stop DMT mainly due to family planning, pregnancy considerations, patient requests, or the side-effect profile of these agents [24]. Moreover, females tend to experience side effects from corticosteroids (e.g. prednisolone or methylprednisolone) more than males [100]. In conclusion, no available phase III clinical trials included prespecified, adequately powered sex-stratified analyses, limiting clear guidance on sex-based variation in treatment response [101]. Parallel to this, for NMOSD and MOGAD, there were insufficient high-quality clinical trials to examine sex differences in treatment response. In addition, differences in response to drugs used for NMOSD, such as inebilizumab, rituximab, and eculizumab, between males and females have not yet been established by robust clinical trials [9]. Similarly, in MOGAD, apparent sex differences in response to immunosuppressive agents such as corticosteroids or mycophenolate are based mainly on clinical experience and cohort studies rather than well-designed randomized clinical trials [9].

During pregnancy, the relapse rate decreases for MS and, to a lesser extent, NMOSD, although findings for NMOSD are less consistent. This phase is followed by a rebound in the relapse rate during the postpartum period, peaking within 3–6 months after delivery [19, 102]. It is also important to consider the effects of maternal immunotherapies on neonates. B-cell-depleting therapies, such as rituximab and inebilizumab, can cross the placenta, especially during the second and third trimesters, leading to transient B-cell lymphopenia in newborns. Although most neonates recover normal B-cell counts within 6 months, these infants may be more susceptible to infections and may require postpartum monitoring and immunization adjustments [103, 104]. Clinicians should weigh the maternal benefits of continuing therapy against potential neonatal immunosuppression and provide appropriate counseling. This change during pregnancy is attributed to pregnancy-induced immunological shifts, including a shift from inflammatory Th1/Th17 immunity to anti-inflammatory Th2 and Treg cells, which enhance peripheral immune tolerance. In addition, pregnancy is associated with reduced B-cell function, lower antibody production, and reduced antigen-presenting cell and autoreactive T-cell activity [105]. In comparison, in the postpartum period, the withdrawal of pregnancy-induced immune tolerance leads to a rebound activation of Th1/Th17 cells, and Treg cells become severely depleted; this is associated with a higher relapse rate [105, 106]. These predictable immunological fluctuations and clinical presentations influence therapeutic decision-making, including when to stop medication before conception and restart it after delivery [107]. Despite a focused, contemporary search of randomized controlled trials over a 26-year period, no trial in MS, NMOSD, or MOGAD formally evaluated sex as an effect modifier on treatment efficacy or safety. Of the 23 articles taken forward to full-text review after title and abstract screening, none reported outcomes stratified by sex or a prespecified male–female subgroup analysis. This points to a striking gap in the design and reporting of demyelinating disease trials. As a result, the trial literature provides little direct evidence on whether treatment effects differ by sex, despite clear biological differences in immune regulation and disease expression.

### Teratogenicity and pregnancy considerations for MS, NMOSD, and MOGAD

When considering teratogenicity, there is variation across MS therapies. Teriflunomide and sphingosine-1-phosphate (S1P) receptor modulators are contraindicated or high risk in pregnancy and should be avoided; teriflunomide requires an accelerated elimination procedure before conception, whereas S1P modulators require drug-specific washout periods prior to pregnancy [108–114]. Withdrawal of S1P modulators is also associated with rebound disease activity, so clinicians should weigh relapse risk against fetal risk and, where appropriate, plan transition to pregnancy-compatible therapies [108, 109, 115]. Moreover, cladribine should also be avoided in pregnancy and requires a 6-month interval after the last dose before conception; however, it may be used as an immune reconstitution strategy before pregnancy to allow a treatment-free period during attempted conception and gestation [108, 109, 116]. In comparison, anti-CD20 therapies are not associated with a clear increase in structural malformations, although placental transfer later in pregnancy can lead to transient neonatal B-cell depletion, with recovery over subsequent months [109, 117, 118]. For NMOSD, monoclonal antibodies such as rituximab, inebilizumab, and eculizumab are increasingly used in selected high-risk pregnancies with careful maternal and fetal monitoring [119–121]. B-cell suppression may affect neonatal immunity, so postpartum vaccination planning and neonatal monitoring are important [119–121]. In MOGAD, corticosteroids and intravenous immunoglobulin remain preferred options, while emerging monoclonal therapies may be considered on a case-by-case basis [120, 121]. Preconception counseling and individualized treatment planning remain essential to optimize maternal and neonatal outcomes [108, 109, 119–121]. Part of this planning should include drug-specific washout advice for therapies with established fetal risk or prolonged pharmacodynamic effects. Accordingly, Table 1 summarizes recommended preconception washout periods for therapies with clinically relevant fetal risk or prolonged pharmacodynamic effects, based on current expert guidance and FDA prescribing information [108–125]. Effective contraception should be advised during these intervals, where applicable. For therapies in which rebound after withdrawal is a major concern, particularly natalizumab and S1P receptor modulators, decisions about discontinuation should be individualized against relapse risk and, when needed, paired with transition to a pregnancy-compatible strategy [109, 115, 126].

**Table 1:** Overview of mechanisms of action, teratogenicity, and pregnancy-related considerations for therapies used in autoimmune demyelinating CNS disorders

Medication	Drug class	Mechanism of action	Pregnancy safety remarks	Major fetal/adverse issues	Recommended washout period prior to conception
Interferon-β (IFN-β) (e.g., Avonex, Betaseron, Rebif, Plegridy)	Cytokine immune modulator	Dampens the inflammation by modulating immune response, e.g., inhibiting T cell migration across the BBB	Safe before conception, during pregnancy and breastfeeding	Rare reports of adverse outcomes such as miscarriage and low birth weight; No established teratogenicity	No fixed preconception washout recommended
Glatiramer acetate (GA) (e.g., Copaxone, Glatopa)	Immune modulator	Induces an anti-inflammatory T-cell response by altering antigen presentation and T-cell polarization, rather than causing broad immunosuppression	Considered safe in pregnancy and breastfeeding	No established teratogenicity	No fixed preconception washout recommended
Natalizumab	α4-integrin inhibitor (Monoclonal antibody)	Prevents leukocyte trafficking and so transmigration into the CNS	In highly active disease, may be continued during pregnancy using extended-interval dosing	Unknown teratogenicity; individual benefit-risk assessment	No fixed preconception washout. If continued during pregnancy, current expert advice is extended-interval dosing until about 30 to 34 weeks gestation
Fingolimod & other S1P modulators (e.g., siponimod, ozanimod)	Sphingosine-1-phosphate receptor modulators	Sequester lymphocytes in lymph nodes preventing their egress and thus their trafficking to the CNS	Contraindicated or high risk**;** requires a washout before conception	Potential teratogenicity suggested from animal studies but limited human studies	Fingolimod: At least 2 months Siponimod: At least 10 days Ozanimod: At least 3 months Ponesimod: At least 7 days
Teriflunomide	Pyrimidine synthesis inhibitor (DHODH inhibitor)	Rapidly inhibits proliferation of T and B cells	Contraindicated/High-Risk**;** teratogenic in animal models	Skeletal abnormalities in preclinical studies	Discontinue and complete accelerated elimination with cholestyramine 8 g TDS for 11 days (or 4 g TDS if not tolerated) or activated charcoal 50 g BD for 11 days Conception should occur only after plasma concentration is < 0.02 mg/L on two tests at least 14 days apart
Cladribine	Purine analog	Selective lymphocyte depletion	Avoid pregnancy; significant washout period recommended	Limited human data, potential fetal risk	At least 6 months after the last dose
Alemtuzumab	Anti-CD52 monoclonal antibody	Causes antibody-dependent cytolytic depletion; immune reconstitution occurs over time	Contraindicated in pregnancy**;** preconception delay recommended	Long-lasting effects on immunity	At least 4 months after the last infusion
Anti-CD20 (Ocrelizumab, Ofatumumab)	B-cell depletion	Targets CD20 on B cells, resulting in B-cell depletion	Limited data; generally advise contraception during and for months after last dose	Potential neonatal B-cell depletion when exposed later in pregnancy	Ocrelizumab: At least 6 months after last dose Ofatumumab: At least 6 months after the last dose

Table 1 illustrates the therapeutic features of drugs used in demyelinating disorders of the CNS.

### Reproductive health and life-course considerations

Across reproductive life stages in women, including menarche, puberty, pregnancy, puerperium, breastfeeding, and menopause, fluctuations in sex hormones influence the risk and activity of autoimmune demyelinating disorders.

Early menarche is associated with an increased risk of MS and an earlier age of onset. Conversely, each additional year in age at menarche has been associated with a nearly 13% reduction in MS risk, although some studies have also linked later menarche to increased disability [127, 128]. Pregnancy is associated with a major reduction in relapse rates, particularly during the third trimester, followed by an increase in relapse risk during the postpartum period [93, 129, 130]. A reduction in relapse rate is less consistently observed in NMOSD during pregnancy because its effector mechanism, which is mainly antibody-mediated complement activation, is less suppressed by pregnancy-related immune tolerance. However, relapse activity in the postpartum period may be as severe as, or more severe than, that seen in MS [131]. In comparison, in MOGAD, a small cohort showed no consistent reduction in relapse risk during pregnancy, while postpartum relapse activity has been reported to a moderate extent; however, the disease is often steroid responsive with good recovery [132].

For breastfeeding and MS disease activity, the evidence remains controversial, with some studies suggesting that breastfeeding decreases relapse rates and others reporting no reduction in disease activity. Regarding menopause, there is also a complex relationship between menopause and MS, as young women with MS may experience earlier menopause, while postmenopausal and perimenopausal women are associated with worsening disability. This is reflected by increasing Expanded Disability Status Scale (EDSS) scores despite a reduction in annual relapse rates during menopause [98]. These disorders do not directly appear to impair fertility; however, high disease activity is often associated with reduced physical readiness for pregnancy. Arabipoor et al. found that ovarian reserve was similar in women with and without MS. Reproductive techniques such as IVF are generally comparable to those used in the general population [133]. There are also conflicting data regarding relapse rates after assisted reproductive technologies (ART), with some studies showing an increase in MS relapse rate after one year and others reporting no increase from baseline [134].

### Psychosocial and quality-of-life implications

Autoimmune demyelinating disorders can cause substantial psychological distress in women, particularly during the young adult years. A recent meta-analysis showed that depression and anxiety were prevalent in 27% and 35% of women with MS, respectively [135]. The depression rate was higher among relapsing–remitting MS, but this was not true for anxiety [135]. In NMOSD, depression and anxiety also appear to be common, although the psychological burden may be shaped by anxiety related to the severity and unpredictability of attacks, where a single relapse can result in permanent blindness or myelopathy [136, 137]. In addition, MS patients are more likely to report fatigue alongside impairment in quality of life, occupational function, and early retirement [138]. MOGAD is associated with cognitive impairment, including verbal learning and memory, information processing speed, attention, visuospatial memory, and verbal fluency, compared with healthy controls [139]. These psychological and cognitive burdens contribute to occupational and social dysfunction and negatively affect women’s life-course responsibilities [140]. Frequent screening, mental health specialist referral, and psychosocial support, alongside immunological disease control, are highly recommended to optimize long-term outcomes. In addition to general psychological distress, certain circumstances uniquely affect women with demyelinating disorders. Pregnancy, postpartum hormonal shifts, family planning, and caregiving responsibilities can exacerbate anxiety, depression, or stress, necessitating specialized mental health interventions. In practice, this may require closer screening during pregnancy and postpartum, multidisciplinary care, and individualized support that accounts for breastfeeding, relapse risk, and caregiving demands. Clinicians should consider these factors when planning therapy, including pharmacologic and non-pharmacologic approaches, to optimize psychological well-being alongside disease management [109, 141, 142].

The clinical consequences of this burden should not be underestimated. In MS, evidence from cohort and pooled analyses suggests higher risks of suicidal ideation, suicide attempts, and suicide mortality than in the general population [143, 144], reinforcing the need for systematic rather than opportunistic psychological screening using validated instruments. Sexual dysfunction, fertility concerns, altered self-image, and fear of transmitting disability to future children represent additional dimensions of distress in women that require direct clinical inquiry beyond standard screening. Where intervention is needed, cognitive behavioral therapy has one of the strongest evidence bases for depression and fatigue in MS [145, 146], and mindfulness-based or exercise-based approaches may be particularly relevant for pregnant or breastfeeding women in whom pharmacological options can be more limited.

To conclude this section, Table 2 provides a side-by-side summary of pathogenesis, immunological, and clinical distinctions across MS, AQP4-IgG-positive NMOSD, and MOGAD with female-specific implications.

**Table 2:** MS vs AQP4-IgG-positive NMOSD vs MOGAD: immunopathology, management, and clinical relevance in women

Domain	MS	AQP4-IgG-positive NMOSD	MOGAD
Immune target / defining antibody	Immune-mediated demyelination; no single defining autoantibody	AQP4-IgG mediated astrocytopathy (seropositivity supports diagnosis)	MOG-IgG-associated inflammatory demyelination
Dominant immune effector profile	T-cell–driven inflammation with B-cell contribution	B-cell/antibody-mediated injury	Antibody-associated demyelination; inflammatory infiltrates common
Typical lesion biology / pathology	CNS demyelination with variable axonal injury; BBB disruption relevant	Astrocyte injury with secondary demyelination; severe attacks can occur	Demyelinating pathology described in MOG-IgG disease
Key diagnostic anchors	MRI dissemination in space/time + CSF oligoclonal bands (context-dependent)	2015 international consensus criteria; AQP4-IgG testing is pivotal	2023 diagnostic criteria performance data; confirm MOG-IgG with appropriate assay
Female: male ratio	2:1 to 3:1	~ 9:1 in AQP4-IgG-positive disease	Sex ratio closer to 1:1 overall (varies by cohort)
Pregnancy & postpartum pattern	Relapse activity usually falls in pregnancy; postpartum relapse risk rises	Pregnancy-related attacks occur; postpartum period remains high risk	Evidence is more heterogeneous; counsel on relapse uncertainty and monitoring
Treatment orientation	Disease-modifying immunotherapy tailored to phenotype and risk	Attack therapy + long-term prevention per NMOSD recommendations	Often steroid-responsive; long-term relapse prevention is individualized
Diagnostic consideration	Assuming all inflammatory demyelination is MS can misdirect treatment choice	Misdiagnosis as MS delays antibody-directed care; confirm AQP4-IgG early	MOGAD can mimic MS/NMOSD; apply criteria and confirm antibody status
Female-specific implications	Discuss contraception, pregnancy timing, and postpartum strategy when selecting DMTs	Preconception/postpartum planning is essential given relapse risk; coordinate obstetric care	Pregnancy planning should be individualized; anticipate steroid use/relapse management needs

*AQP4-IgG* aquaporin-4 immunoglobulin G, *BBB* blood–brain barrier, *CNS* central nervous system, *CSF* cerebrospinal fluid, *DMT* disease-modifying therapy, *MRI* magnetic resonance imaging, *MOG-IgG* myelin oligodendrocyte glycoprotein immunoglobulin G, *MOGAD* myelin oligodendrocyte glycoprotein antibody-associated disease, *MS* multiple sclerosis, *NMOSD* neuromyelitis optica spectrum disorder

## Conclusion

Women are affected more often than men across demyelinating CNS disorders, most notably in AQP4-IgG-positive NMOSD, followed by MS; in MOGAD, sex ratios are closer to 1:1 overall, with a weak female predominance reported in some cohorts. This skewed distribution appears to stem from multiple interconnected factors: sex-specific immunological pathways, hormonal modulation of immune function, and genetic susceptibility patterns that collectively influence disease onset, clinical course, and treatment outcomes. Female patients demonstrate enhanced immune reactivity, characterized by increased CD4 + T cell and B-cell activity, resulting in higher antibody production. These immunological differences present clinical challenges that pertain specifically to women, who consequently are more likely to develop relapsing forms of MS and experience higher relapse rates of the disease, especially during periods of hormonal fluctuation.

In MS, relapse activity usually falls during pregnancy; this also occurs in NMOSD, but the relapse risk reduction is smaller. After delivery, relapse risk commonly rebounds, peaking in the first 3 months postpartum. Such changes necessitate careful alterations to treatment plans, such as determining appropriate timing for discontinuing medications prior to conception and resuming them at the appropriate time following childbirth. Anti-CD20 therapies are not linked to structural malformations, but exposure late in pregnancy can temporarily lower neonatal B-cell counts. For patients with high-risk NMOSD, agents such as azathioprine and rituximab are used, though data on their safety during pregnancy remain limited. These patients could benefit greatly from individualized counseling and close collaboration with maternal–fetal medicine specialists to achieve optimal outcomes.

Our review of RCTs conducted between 2000 and 2026 found no sex-stratified analyses reporting treatment efficacy or safety in MS, NMOSD, or MOGAD. This absence of sex-disaggregated reporting limits how confidently clinicians can individualize treatment for women across key life stages, particularly when decisions are shaped by pregnancy planning, postpartum relapse risk, and adverse-effect burden. Future RCTs should prespecify sex-stratified outcomes and report efficacy and adverse events in sex-specific subgroups. Depression, anxiety, fatigue, and cognitive impairment are common in women with demyelinating conditions and often worsen during hormonal transitions, which can also coincide with an increased risk of disease flares. Consequently, female patients require care that extends beyond relapse prevention. Routine screening for psychological symptoms and clear pathways to psychosocial support are not part of standard care. This is likely due to the evidence base remaining too limited to guide consistent clinical implementation.

Despite strong evidence that demyelinating disorders are more common in women than in men, there is insufficient evidence to confidently explain the reasons for this gap or how sex-specific factors shape demyelination and, in turn, affect clinical course and management across a woman’s lifespan. To help clarify mechanisms of demyelination and inform stage-specific management in women, future studies are needed that track reproductive stage and hormone exposure alongside immunological and imaging markers.

## Supplementary Information

Below is the link to the electronic supplementary material.

Supplementary file1 (DOCX 21 KB)

## Data Availability

Data sharing is not applicable to this article as no datasets were generated or analyzed during the current study.
