# Supplementary material for: Demyelinating disorders in women: epidemiology, immunology, and clinical implications across MS, NMOSD, and MOGAD
Source: J Neurol. 2026 Apr 27;273(5):290. doi: 10.1007/s00415-026-13831-z (PMC13121431; doi:10.1007/s00415-026-13831-z)
Supplement: Supplementary file 1 — Supplementary file1 (DOCX 21 KB) [file 415_2026_13831_MOESM1_ESM.docx]

**Online Resource 1, Table 1: Search strategy used in PubMed and Scopus databases:**

| **Search Strategy- Pubmed** | **Results** |
| --- | --- |
| ("multiple sclerosis"[Title/Abstract] OR "multiple sclerosis"[MeSH Terms]) AND ("randomized controlled trial"[Publication Type] OR "randomized"[Title/Abstract] OR "randomised"[Title/Abstract] OR "RCT"[Title/Abstract]) AND ("sex differences"[Title/Abstract] OR "gender differences"[Title/Abstract] OR "sex-specific"[Title/Abstract] OR "male"[Title/Abstract] OR "female"[Title/Abstract] OR "women"[Title/Abstract] OR "men"[Title/Abstract]) AND (treatment[Title/Abstract] OR therapy[Title/Abstract] OR medication[Title/Abstract] OR "disease modifying"[Title/Abstract] OR "DMT"[Title/Abstract]) | 158 results |
| ("neuromyelitis optica spectrum disorder"[Title/Abstract] OR "NMOSD"[Title/Abstract] OR "neuromyelitis optica"[Title/Abstract]) AND ("randomized controlled trial"[Publication Type] OR "randomized"[Title/Abstract] OR "randomised"[Title/Abstract] OR "RCT"[Title/Abstract]) AND ("sex differences"[Title/Abstract] OR "gender differences"[Title/Abstract] OR "male"[Title/Abstract] OR "female"[Title/Abstract] OR "women"[Title/Abstract] OR "men"[Title/Abstract]) AND (treatment[Title/Abstract] OR therapy[Title/Abstract] OR medication[Title/Abstract]) | 2 results |
| ("myelin oligodendrocyte glycoprotein antibody disease"[Title/Abstract] OR "MOGAD"[Title/Abstract] OR "MOG antibody"[Title/Abstract]) AND ("randomized controlled trial"[Publication Type] OR "randomized"[Title/Abstract] OR "randomised"[Title/Abstract] OR "RCT"[Title/Abstract]) AND ("sex differences"[Title/Abstract] OR "gender differences"[Title/Abstract] OR "men"[Title/Abstract] OR "male"[Title/Abstract] OR "female"[Title/Abstract] OR "women"[Title/Abstract]) AND (treatment[Title/Abstract] OR therapy[Title/Abstract] OR medication[Title/Abstract]) | 0 Results |
| **Search Strategy for Scopus Database** | **Results** |
| TITLE-ABS-KEY ( "myelin oligodendrocyte glycoprotein antibody disease" OR MOGAD OR "MOG antibody" ) AND TITLE-ABS-KEY ( "randomized controlled trial" OR randomized OR randomised OR RCT ) AND TITLE-ABS-KEY ( "sex differences" OR "gender differences" OR male OR female OR women OR men ) AND TITLE-ABS-KEY ( treatment OR therapy OR medication ) | 15 results |
| TITLE-ABS-KEY ( "multiple sclerosis" OR MS ) AND TITLE ( female OR women ) AND TITLE-ABS-KEY ( treatment OR medication OR "disease modifying" ) AND ( LIMIT-TO ( DOCTYPE , "ar" ) ) | 1045 results |
| TITLE-ABS-KEY ( "neuromyelitis optica spectrum disorder" OR NMOSD OR "neuromyelitis optica" ) AND TITLE-ABS-KEY ( "randomized controlled trial" OR randomized OR randomised OR RCT ) AND TITLE-ABS-KEY ( "sex differences" OR "gender differences" OR male OR female OR women OR men ) AND TITLE-ABS-KEY ( treatment OR therapy OR medication ) AND ( LIMIT-TO ( DOCTYPE , "ar" ) ) | 77 results |

**Online Resource 1, Table 2: The references and resources of female-to-male ratio for neuromyelitis optica spectrum disorders (NMOSD) by country - *case definition and AQP4-IgG status vary by study.***

| Country | F : M ratio | Reference |
| --- | --- | --- |
| Algeria | 3 | Daoudi, S., & Bouzar, M. (2016). Neuromyelitis optica spectrum disorders in Algeria: a preliminary study in the region of Tizi Ouzou. Multiple sclerosis and related disorders, 6, 37-40. |
| Australia | 6 | Bukhari W, Prain KM, Waters P et al (2017) Incidence and preva- lence of NMOSD in Australia and New Zealand. J Neurol Neuro- surg Psychiatry 88:632–638 |
| Austria | 7 | Aboul-Enein F, Seifert-Held T, Mader S, Kuenz B, Lutterotti A, Rauschka H, et al. Neuromyelitis optica in Austria in 2011: to bridge the gap between neuroepidemiological research and practice in a study population of 8.4 million people. PLoS ONE. (2013) 8:e79649. doi: 10.1371/journal.pone.0079649 |
| Brazil | 5 | Petersen, G., Inzunza, A., & Levy, M. (2025). LATAM-Panorama-NMOSD Epidemiological, Clinical, and Paraclinical Features of Neuromyelitis Optica Spectrum Disorder in Latin America and the Caribbean A Systematic Review and Meta-analysis. Multiple Sclerosis and Related Disorders, 106604. |
| Canada | 5 | Arnett, S., Chew, S. H., Leitner, U., Hor, J. Y., Paul, F., Yeaman, M. R., ... & Broadley, S. A. (2024). Sex ratio and age of onset in AQP4 antibody-associated NMOSD: a review and meta-analysis. Journal of neurology, 271(8), 4794-4812. |
| Chile | 7 | Henríquez, K., Molt, F., Gajardo, J., Cortés, B., & Ramirez-Santana, M. (2022). Sociodemographic and clinical characteristics of people with multiple sclerosis and neuro-myelitis optica spectrum disorder in a central northern region of Chile: A prevalence study. Multiple Sclerosis and Related Disorders, 61, 103750. |
| China | 4.7 | Tian, D. C., Li, Z., Yuan, M., Zhang, C., Gu, H., Wang, Y., & Shi, F. D. (2020). Incidence of neuromyelitis optica spectrum disorder (NMOSD) in China: a national population-based study. The Lancet Regional Health–Western Pacific, 2. |
| Denmark | 4.5 | Papp V, Illes Z, Magyari M, Koch-Henriksen N, Kant M, Pfleger CC, et al. Nationwide prevalence and incidence study of neuromyelitis optica spectrum disorder in Denmark. Neurology. (2018) 91:e2265– 75. doi: 10.1212/WNL.0000000000006645 |
| Ecuador | 4.4 | Petersen, G., Inzunza, A., & Levy, M. (2025). LATAM-Panorama-NMOSD Epidemiological, Clinical, and Paraclinical Features of Neuromyelitis Optica Spectrum Disorder in Latin America and the Caribbean A Systematic Review and Meta-analysis. Multiple Sclerosis and Related Disorders, 106604. |
| Egypt | 5.6 | Musubire, A. K., Derdelinckx, J., Reynders, T., Meya, D. B., Bohjanen, P. R., Cras, P., & Willekens, B. (2021). Neuromyelitis optica spectrum disorders in Africa: a systematic review. Neurology: Neuroimmunology & Neuroinflammation, 8(6), e1089. |
| France | 3.1 | Arnett, S., Chew, S. H., Leitner, U., Hor, J. Y., Paul, F., Yeaman, M. R., ... & Broadley, S. A. (2024). Sex ratio and age of onset in AQP4 antibody-associated NMOSD: a review and meta-analysis. Journal of neurology, 271(8), 4794-4812. |
| Germany | 5 | Borisow, N., Kleiter, I., Gahlen, A., Fischer, K., Wernecke, K. D., Pache, F., ... & NEMOS (Neuromyelitis Optica Study Group). (2017). Influence of female sex and fertile age on neuromyelitis optica spectrum disorders. Multiple Sclerosis Journal, 23(8), 1092-1103. |
| Hungary | 8.8 | Papp V, Iljicsov A, Rajda C, Magyari M, Koch-Henriksen N, Petersen T, et al. A population-based epidemiological study of neuromyelitis optica spectrum disorder in hungary. Eur J Neurol. (2020) 27:308–17. doi: 10.1111/ene.14079 |
| India | 1.2 | Pandit L, Kundapur R. Prevalence and patterns of demyelinating central nervous system disorders in urban Mangalore, South India. Mult Scler. (2014) 20:1651–3. doi: 10.1177/1352458514521503 |
| Iran | 5 | Eskandarieh S, Nedjat S, Azimi AR, Moghadasi AN, Sahraian MA. Neuromyelitis optica spectrum disorders in Iran. Mult Scler Relat Disord. (2017) 18:209–12. doi: 10.1016/j.msard.2017.10.007 |
| Italy | 6 | Arnett, S., Chew, S. H., Leitner, U., Hor, J. Y., Paul, F., Yeaman, M. R., ... & Broadley, S. A. (2024). Sex ratio and age of onset in AQP4 antibody-associated NMOSD: a review and meta-analysis. Journal of neurology, 271(8), 4794-4812. |
| Japan | 6.4 | Miyamoto K, Fujihara K, Kira JI, Kuriyama N, Matsui M, Tamakoshi A, et al. Nationwide epidemiological study of neuromyelitis optica in Japan. J Neurol Neurosurg Psychiatry. (2018) 89:667–8. doi: 10.1136/jnnp-2017-317321 |
| Malaysia | 14 | Hor JY, Lim TT, Chia YK, Ching YM, Cheah CF, Tan K, et al. Prevalence of neuromyelitis optica spectrum disorder in the multi- ethnic penang island, malaysia, and a review of worldwide prevalence. Mult Scler Relat Disord. (2018) 19:20–4. doi: 10.1016/j.msard.2017. 10.015 |
| Mexico | 4 | Mireles-Ramírez, M. A., Velázquez-Brizuela, I. E., Sánchez-Rosales, N., Márquez-Pedroza, Y., Hernandez-Preciado, M. R., & Ortiz, G. G. (2022). The prevalence, incidence, and clinical assessment of neuromyelitis optica spectrum disorder in patients with demyelinating diseases. Neurología (English Edition). |
| Morocco | 3.6 | Bennis, A., El Otmani, H., Benkirane, N., Harrizi, I., El Moutawakil, B., Rafai, M. A., & Slassi, I. (2019). Clinical course of neuromyelitis optica spectrum disorder in a moroccan cohort. Multiple sclerosis and related disorders, 30, 141-148. |
| Netherlands | 4.9 | Van Pelt ED, Wong YY, Ketelslegers IA, Siepman DA, Hamann D, Hintzen RQ. Incidence of AQP4-IgG seropositive neuromyelitis optica spectrum disorders in the Netherlands: about one in a million. Mult Scler Exp Transl Clin. (2016) 2:2055217315625652. doi: 10.1177/2055217315625652 |
| Peru | 6 | Lana-Peixoto, M. A., Talim, N. C., Pedrosa, D., Macedo, J. M., & Santiago-Amaral, J. (2021). Prevalence of neuromyelitis optica spectrum disorder in Belo Horizonte, Southeast Brazil. Multiple Sclerosis and Related Disorders, 50, 102807. |
| Portugal | 4 | Arnett, S., Chew, S. H., Leitner, U., Hor, J. Y., Paul, F., Yeaman, M. R., ... & Broadley, S. A. (2024). Sex ratio and age of onset in AQP4 antibody-associated NMOSD: a review and meta-analysis. Journal of neurology, 271(8), 4794-4812. |
| Russia | 4.26 | Hor, J. Y., Asgari, N., Nakashima, I., Broadley, S. A., Leite, M. I., Kissani, N., ... & Fujihara, K. (2020). Epidemiology of neuromyelitis optica spectrum disorder and its prevalence and incidence worldwide. Frontiers in neurology, 11, 501. |
| Senegal | 3 | Musubire, A. K., Derdelinckx, J., Reynders, T., Meya, D. B., Bohjanen, P. R., Cras, P., & Willekens, B. (2021). Neuromyelitis optica spectrum disorders in Africa: a systematic review. Neurology: Neuroimmunology & Neuroinflammation, 8(6), e1089. |
| South Korea | 4.7 | Lee HL, Kim JY, Seok JM, Hong YH, Lim NG, Shin HY, et at. Prevalence and incidence of neuromyelitis optica spectrum disorder in Korea: population based study. J Korean Med Sci. (2020) 35:e115. doi: 10.3346/jkms.2020.35.e115 |
| Spain | 3.1 | Arnett, S., Chew, S. H., Leitner, U., Hor, J. Y., Paul, F., Yeaman, M. R., ... & Broadley, S. A. (2024). Sex ratio and age of onset in AQP4 antibody-associated NMOSD: a review and meta-analysis. Journal of neurology, 271(8), 4794-4812. |
| Sweden | 2.8 | Jonsson DI, Sveinsson O, Hakim R, Brundin L. Epidemiology of NMOSD in Sweden from 1987 to 2013: a nationwide population-based study. Neurology. (2019) 93:e181–9. doi: 10.1212/WNL.0000000000 007746 |
| Turkey | 8.2 | Taşkıran, E., Öztürk, B., Demir, S., Ata, N., Ülgü, M. M., Birinci, Ş., ... & Kürtüncü, M. (2025). Prevalence and incidence of neuromyelitis optica spectrum disorder in Türkiye: A nationwide epidemiologic study. Multiple Sclerosis and Related Disorders, 97, 106383. |
| United Kingdom | 3 | Jacob A, Panicker J, Lythgoe D, Elsone L, Mutch K, Wilson M, et al. The epidemiology of neuromyelitis optica amongst adults in the merseyside county of United Kingdom. J Neurol. (2013) 260:2134– 7. doi: 10.1007/s00415-013-6926-y |
| United States of America | 3.5 | Briggs, F. B., & Shaia, J. (2024). Prevalence of neuromyelitis optica spectrum disorder in the United States. Multiple Sclerosis Journal, 30(3), 316-324. |
| Venezuela | 4 | de Castillo Ibis, S., Omaira, M., Arnoldo, S., Elizabeth, A., Sandra, M., Carlota, C. M., ... & Hernandez, F. (2021). Epidemiological findings of neuromyelitis optica spectrum disorders in a Venezuelan study. Multiple Sclerosis and Related Disorders, 47, 102652. |
